# Enhanced Nutraceutical Properties of Extra Virgin Olive Oil Extract by Olive Leaf Enrichment

**DOI:** 10.3390/nu15051073

**Published:** 2023-02-21

**Authors:** Doretta Cuffaro, Simone Bertini, Marco Macchia, Maria Digiacomo

**Affiliations:** 1 Department of Pharmacy, University of Pisa, via Bonanno 6, 56126 Pisa, Italy; 2 Interdepartmental Research Center “Nutraceuticals and Food for Health”, University of Pisa, 56100 Pisa, Italy

**Keywords:** extra virgin olive oil, olive leaves, polyphenols, antioxidant activity, anti-inflammatory activity, EVOO enrichment

## Abstract

(1) Background: Nowadays, the health-promoting properties of extra virgin olive oil (EVOO), including the antioxidant and anti-inflammatory actions, are well recognized and mainly attributed to the different polyphenols, such as oleocanthal and oleacein. In EVOO production, olive leaves represent a high value by-product, showing a wide spectrum of beneficial effects due to the presence of polyphenols, especially oleuropein. Here we report the study of olive leaf extract (OLE)-enriched EVOO extracts, obtained by adding different percentages of OLE to EVOO in order to ameliorate their nutraceutical activities. (2) Methods: The polyphenolic content of the EVOO/OLE extracts was analyzed by HPLC and the Folin-Ciocalteau assay. For further biological testing, an 8% OLE-enriched EVOO extract was chosen. Therefore, antioxidant effects were evaluated by three different methods (DPPH, ABTS, and FRAP), and the anti-inflammatory properties were assessed in terms of cyclooxygenase activity inhibition. (3) Results: The antioxidant and anti-inflammatory profiles of the new EVOO/OLE extract are significantly improved compared to those of EVOO extract; (4) Conclusions: The combination of OLE and EVOO extract can lead to an extract enriched in terms of bioactive polyphenols and endowed with better biological properties than the singular EVOO extract. Therefore, it may represent a new complement in the nutraceutical field.

## 1. Introduction

The Mediterranean diet plays a salient role in the primary prevention of chronic disorders, as documented by several studies [1,2]. The healthy properties of the Mediterranean diet are correlated with high consumption of extra virgin olive oil (EVOO), which is rich in polyphenols. In EVOO, the high content of phenolic compounds exerts, among other things, antioxidant, anti-inflammatory, anti-cancer, and antimicrobial effects [3,4,5]. Nevertheless, polyphenols are contained in EVOO in small quantities, less than 2%, as the main constituents of EVOO are triglycerides. Among EVOO polyphenols, the simple phenols such as tyrosol and hydroxytyrosol and the secoiridoids can be distinguished. Secoiridoids constitute the largest family of polyphenols in EVOO, and the main representative compounds are oleacein and oleocanthal, endowed with important nutraceutical properties [6,7]. Beauchamp et al. reported the anti-inflammatory activity of oleocanthal as comparable to that of ibuprofen, a known non-steroidal anti-inflammatory drug (NSAID) [8]. Moreover, oleocanthal showed other important biological activities, such as anticancer activity [9,10], an anti-Alzheimer effect [11,12], and a protective role in arthropathy [13] and cardiovascular diseases [14]. Oleacein, similarly to oleocanthal, decreases cyclooxygenase activity, thereby reducing inflammation [15,16], and is responsible for the anti-sclerotic effect attributed to EVOO [17]. Furthermore, oleacein revealed in vitro activity against some types of cancer [18] and an anti-estrogenic effect [19].

In the last decade, the EVOO industry’s production has grown considerably, causing a significant environmental impact in terms of waste generation. Olive leaves are the primary source of waste generation, accounting for 10% of harvested olives by weight. Olive leaves are an abundant but unavoidable waste that accumulates in high amounts during the tree-pruning process of olive trees [20]. It is estimated that for every liter of produced EVOO, 6.23 kg of pruning residues (consisting of leaves and branches) are generated [21], and for example, in Spain every year an average of 750,000 tons of olive leaves have been gathered [22]. Considering the huge quantities of leaves, many efforts to use them and obtain energy or nutraceutical molecules from them have been made. Likewise, olive leaves represent the main site of plant metabolism, where photosynthesis takes place, generating a valuable source of primary and secondary plant-derived products. Therefore, bioactive compounds, including polyphenols such as oleuropein, hydroxytyrosol, and verbascoside, and flavonoids such as luteolin and apigenin, are amply present in olive leaves. Olive leaves have been shown to have health benefits such as antioxidant, antimicrobial, and anti-atherosclerosis effects, owing to the presence of oleuropein [7,23,24]. Indeed, in vitro and in vivo studies underlined a plethora of properties for oleuropein, such as the antioxidant, antimicrobial, antifungal, anti-tumoral, hypolipidemic, and especially hypotensive, anticancer, and cardioprotective actions [25,26,27,28,29]. Moreover, the anti-inflammatory effect of oleuropein in terms of pro-inflammatory agent inhibition, and especially cyclooxygenase (COX) inhibition, is consistently reported by several studies [30,31]. Oleuropein content is significantly high in olive leaves, in sharp contrast to EVOO, where it is present only marginally. Therefore, enriching the polyphenol content of EVOO extract by adding olive leaf extract (OLE) as a source of oleuropein may promote synergistic interactions between nutraceutical polyphenols, increasing EVOO’s health promoting properties. In fact, it is known that the combination of extracts can lead to better chemotherapeutic and chemopreventive effects than either extract used alone, thanks to synergistic interactions between the components [32].

**Figure 1:**
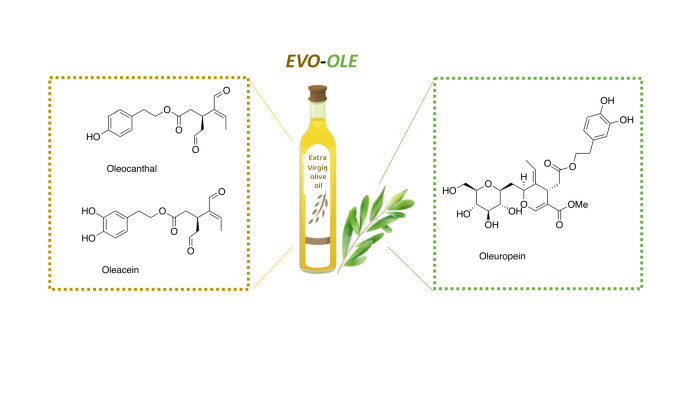
Chemical structures of the principal polyphenols of EVOO and OLE extracts.

Starting from these evidences, in this work we propose the study of new EVOO/OLE extracts obtained by adding an oleouropein-rich OLE to EVOO extract in different ratios, with the aim of increasing the polyphenol content and ameliorating the biological activities of EVOO. In the literature, some studies investigating the variation in polyphenolic composition after the addition of olive leaves to EVOO have already been reported, focusing mainly on the extraction method, composition, and quality of EVOO [33,34]. In this work, our attention is focused on the evaluation of how the nutraceutical properties of EVOO could be affected by the addition of OLE. In particular, we investigate the antioxidant and anti-inflammatory properties of EVOO/OLE 8% extract, which presents a high content of some of the most important nutraceutical polyphenols: oleocanthal, oleacein, and oleuropein (Figure 1). This new approach might be a proper strategy to valorize olive leaves as a sustainable alternative use, converting them into higher-value by-products. This study well aligns with the purposes of the Recovery and Resilience Plan regarding the re-evaluation of biowastes derived from the food supply chain, which significantly affect the environment.

## 2. Materials and Methods

### 2.1. Chemicals and Standards

Solvents used for extraction procedures and HPLC analyses were purchased from Merck (Merck srl, Milan, Italy). The pure standards oleocanthal and oleacein were obtained through EVOO extraction and purification using the method described in our previous work [35]. The following commercial compounds are used as analytical standards: oleuropein, *p*-hydroxyphenylacetic acid, and Trolox, purchased from Merck (Merck srl, Milan, Italy); tyrosol and hydroxytyrosol purchased from TCI (Zwijndrecht, Belgium); and luteolin-7-*O*-glucoside and apigenin-7-*O*-glucoside purchased from Extrasynthese (Lyon, France). Folin–Ciocalteu reagent (FCR), 2,2-diphenyl-1-picrylhydrazyl (DPPH) 2,2′-azino-bis(3-ethylbenzothiazoline-6-sulfonic acid (ABTS), and 2,4,6-Tris(2-pyridyl)-s-triazine (TPTZ) were purchased from Merk (Darmstadt, Germany).

### 2.2. Preparation of Samples

#### 2.2.1. Extra Virgin Olive Oil Extract

An EVOO sample (Moraiolo, Frantoio, and Leccino varieties) produced in the 2020/2021 crop season was used as an EVOO sample. The EVOO extract was prepared as previously described [10]. Briefly, to the EVOO sample (3 g) n-hexane (12 mL) and acetonitrile (15 mL) were added. After homogenization in a vortex mixer for 30 s and a rotary shaker for 30 min, the mixture was centrifugated at 4000 rpm for 5 min. Then the acetonitrile phase was separated and evaporated to afford EVOO extract.

#### 2.2.2. Olive Leaf Extract

Olive leaves were derived from Olivastra seggianese groves located at CNR-IVALSA, Follonica (GR), Italy. The collection was performed manually in September 2019 and stored at 25 °C. After harvesting, 20 g of leaves were put in liquid nitrogen and crushed manually. Afterwards, water was added to the powdered leaves and the solution was sonicated and mixed by vortex. Then the solution was centrifugated at 4000 rpm for 5 min at 25 °C, and the water phase was filtered and freeze-dried, affording OLE.

#### 2.2.3. Extra Virgin Olive Oil/Olive Leaf Extract

A solution of EVOO extract in MeOH (20 mg/mL) and of OLE in water (2 mg/mL) have been prepared. 8%, 4%, 2%, and 1% of OLE water solution have been added to a methanolic EVOO solution, obtaining the corresponding EVOO/OLE extracts.

### 2.3. Determination of Total Phenolic Content

The total phenolic content (TPC) of the extracts was evaluated using FCR as previously described [36]. Briefly, 2.5 g of the EVOO sample were dissolved in 5 mL of n-hexane and subsequently extracted with 5 mL of MeOH (80% *v*/*v*). The methanolic phase was collected, obtaining 10 mL of methanolic extract. EVOO-OLE 8% and OLE sample extracts have been prepared as reported in Section 2.2.2 and Section 2.2.3, respectively. After evaporation, the resulting dry extract was dissolved in 1 mL of a solution of methanol (80% *v*/*v*).

A total of 0.25 mL of FCR and 1.5 mL of Na_2_CO_3_ (20% *w*/*v*) were added to 1 mL of the methanolic solution in a volumetric flask, and then distilled water was added up to 10 mL. The solution was incubated for 45 min at 25 °C, and then the absorbance was read at λ = 725 nm. TPC was calculated using a gallic acid calibration curve (2.5–40.0 μg/mL) and expressed as mg of gallic acid (GA) equivalent/kg of sample (ppm). mg gallic acid equivalent). Analyses were performed in triplicate, and the mean value was calculated for each sample.

### 2.4. Analysis of Phenolic Compounds

HPLC analysis of samples, prepared as illustrated in Section 2.3, was carried out using a slightly revised method reported in our previous studies [35,37]. The extracts were injected as a mixture of MeOH/H_2_O (1:1 *v*/*v*) in a Shimadzu HPLC Nexera series (model CBM-40D), which consisted of a binary pump (LC-40D XR), a degassing unit (DGU-405), and a diode array detector (SPD-M40) (Shimadzu, OR, USA). The data processing was performed on the Shimadzu LabSolutions software LC-GC. HPLC analysis was performed using a Phenomenex Gemini reverse-phase C18 column (250 × 4.6 mm, 5 µm particle size; Phenomenex, Castel Maggiore, Italy). A *p*-hydroxyphenylacetic acid was chosen as an internal standard. In the mobile phase, a mixture of H_2_O/AcOH (97.5:2.5 *v*/*v*) (A) and ACN/MeOH (1:1 *v*/*v*) (B) was used. The linear gradient progressed from 5% (B) to 30% (B) in 45 min; it changed to 70% (B) during 20 min (65 min total time); in 5 min it changed to 80% (B) (70 min total time); it remained at 80% (B) for 15 min (85 min total time); it changed to 100% (B) in 5 min (90 min total time); after re-equilibration for 5 min (95 min total time) to initial composition, it remained at 5% (B) for 10 min (105 min total time). The flow rate was 1 mL/min, and the injected volume was 20.0 μL.

### 2.5. DPPH• Radical Scavenging Activity

The antioxidant activity of OLE, EVOO, and EVOO/OLE extracts was assessed through the DPPH• free radical scavenging assay, which was modified slightly from Brand–Willians [38]. A methanol solution of DPPH• (40.0 μg/mL) was added to the samples solubilized in MeOH at different concentrations (1 mg/mL–6 mg/mL for EVOO extract, 0.3 mg/mL–1 mg/mL for EVOO/OLE extract, and 0.3–0.025 mg/mL for OLE). After 45 min of incubation at room temperature and in the dark, the absorbance was read at 517 nm in a SPECTROstarNano (200–1000 nm) UV/Vis spectrophotometer (BMG Labtec, Germany). MeOH was used as a blank, and Trolox^®^ was used as a positive control (0.015–0.0005 mg/mL) and treated under the same conditions as the samples. The percent of antioxidant activity (%AA) was calculated according to the following formula:

%AA = (Abs_DPPH_) − (Abs_sample_)/Abs_DPPH_ × 100

Abs_DPPH_ = absorbance of DPPH solution, subtracted from the absorbance of MeOH

Abs_sample_ = absorbance of DPPH solution including the test compound subtracted from the absorbance of test compound solution without DPPH.

The results were expressed as the efficient inhibitory concentration of antioxidants necessary to decrease the initial DPPH concentration by 50% (EC_50_). EC_50_ has been calculated by linear regression, as already reported [39]. All experiments were performed in triplicate.

### 2.6. ABTS•+ Radical Scavenging Activity

The free radical scavenging activity of OLE and EVO, EVO-OLE 8% extracts was assessed by using ABTS radical cation decolouration assay, which was modified from the protocol reported by Pellegrini et al. [40]. Briefly, an ABTS solution was prepared by mixing an aqueous solution of ABTS (7 mM) with potassium persulfate (2.45 mM) in a 1:1 ratio. The solution was incubated for 12 h in the dark at room temperature, then it was diluted with water to an absorbance of 0.7 at 750 nm. 190 µL of ABTS solution were mixed with 10 µL of sample dissolved in EtOH. The solution was incubated for 5 min at room temperature, and the final absorbance was read at 734 nm in a SPECTROstarNano (200–1000 nm) UV/Vis spectrophotometer (BMG Labtec, Germany). Calculations were performed to evaluate the percentage of inhibition of the ABTS radical cation as follows:

% scavenging ability = (Abs_ABTS_ − Abs_sample_)/Abs_ABTS_ × 100

Abs_ABTS_ = the absorbance of the ABTS solution

Abs_sample_ = the absorbance of the ABTS solution containing the test compound. (1)

The percentage of scavenging ability was calculated against the sample concentration to give the efficient concentration to decrease the initial ABTS concentration by 50% (EC_50_). All experiments were performed in triplicate.

### 2.7. Ferric Reducing Antioxidant Power Assay

The method described by Borges et al. [41], was used to assess the antioxidant activity. Ferric Reducing Antioxidant Power (FRAP) assay measures the ferric-reducing ability of a sample in an acid medium (pH 3.6) through the formation of a specific blue color as the ferric tripyridyltriazine (Fe^3+^–TPTZ) complex, caused by reduction to the ferrous (Fe^2+^) form. The FRAP reagent was obtained by mixing acetate sodium buffer (0.3 M) at pH 3.6, ferric chloride (20 mM), and TPTZ (10 mM) in HCl (40 mM) in a ratio of 10:1:1. 20 µL of extracts (OLE, EVO, or EVO/OLE 8%) were mixed with FRAP solution (280 µL), and the mixture was incubated at 37 °C for 30 min. The absorbance of the reaction mixture was read at 595 nm in a SPECTROstarNano (200–1000 nm) UV/Vis spectrophotometer (BMG Labtec, Ortenberg, Germany). The calibration curve was built using different concentrations of Trolox^®^ (0.01–0.2 mg mL^−1^), and the results are expressed as mmol of Trolox equivalents per kg of the sample. All experiments were performed in triplicate.

### 2.8. Cyclooxygenase Enzyme Inhibitory Assay

The ability of OLE, EVOO, and EVOO/OLE extracts to inhibit COX-1 and COX-2 was evaluated using a COX-1 (ovine) and COX-2 (human)-inhibitor screening assay (kit No. 701050 from Cayman Chemical Co. Michigan, USA), following the manufacturer’s protocols. An initial COX-1/COX-2 inhibitory evaluation test was performed at 225 µg/mL for all the extracts (OLE, EVO, and EVO/OLE 8%). Then, increasing concentrations of each sample (45–225 µg/mL) have been tested. Arachidonic acid at 1.1 mM was the substrate, and ibuprofene was used as a control. The peroxidase activity was examined colorimetrically at 590 nm after an incubation of 120 min at room temperature using a SPECTROstarNano (200–1000 nm) UV/Vis spectrophotometer (BMG Labtec, Germany). All tests were performed three times. The percent (%) inhibition of COX-1 and COX-2 is derived from the following formula:

% inhibition = (EAA − AIA)/EAA × 100

EAA = Enzyme test activity absorbance

AIA = Activity inhibition test absorbance.(2)

Results were expressed as % of inhibition or inhibitory concentration at 50% (IC_50_) calculated by least-squares regression analysis of inhibition versus concentration, as already reported [8,42,43]. All experiments were performed in triplicate.

### 2.9. Statistical Analysis

Data were presented as the mean ± standard deviation of three independent experiments. Graphpad 9.0 has been used to investigate the statistical differences among results. A one-way analysis of variance (ANOVA) was applied to determine the differences between samples, and Turkey’s multiple-comparison test was used as a post hoc comparison of the means. A denoting significance was accepted for *p* < 0.05.

## 3. Results

### 3.1. Phenolic Compound Content of Extracts

The phenolic contents of EVOO, OLE, and EVOO/OLE have been evaluated by qualitative and quantitative HPLC analysis. The results are reported in Table 1. From HPLC analysis of EVOO, it emerged that it contains high amounts of oleacein (11.05 mg/g EVOO extract) and oleocanthal (20.97 mg/g EVOO extract) and a small quantity of hydroxytyrosol and tyrosol (0.72 and 1.02 mg/g EVOO extract, respectively) as expected for a fresh EVOO [37]. Regarding OLE, the most representative phenolic compound is oleuropein (35.58 mg/g OLE), followed by luteolin-7-*O*-glucoside and apigenin-7-*O*-glucoside (6.70 and 1.80 mg/g OLE, respectively), as confirmed by the literature [44]. The enriched EVOO/OLE extracts maintained high quantities of oleocanthal and oleacein with increasing amounts of oleuropein and other polyphenols usually not present in EVOO, such as luteolin-7-*O*-glucoside and apigenin-7-*O*-glucoside. EVOO/OLE 8% presented the best profile in terms of phenolic compound content, with a balanced amount of oleacein (10.84 mg/g), oleocanthal (19.03 mg/g), and oleuropein (8.46 mg/g). Moreover, luteolin-7-*O*-glucoside, apigenin-7-*O*-glucoside, tyrosol, and hydroxytyrosol are present.

**Figure 2:**
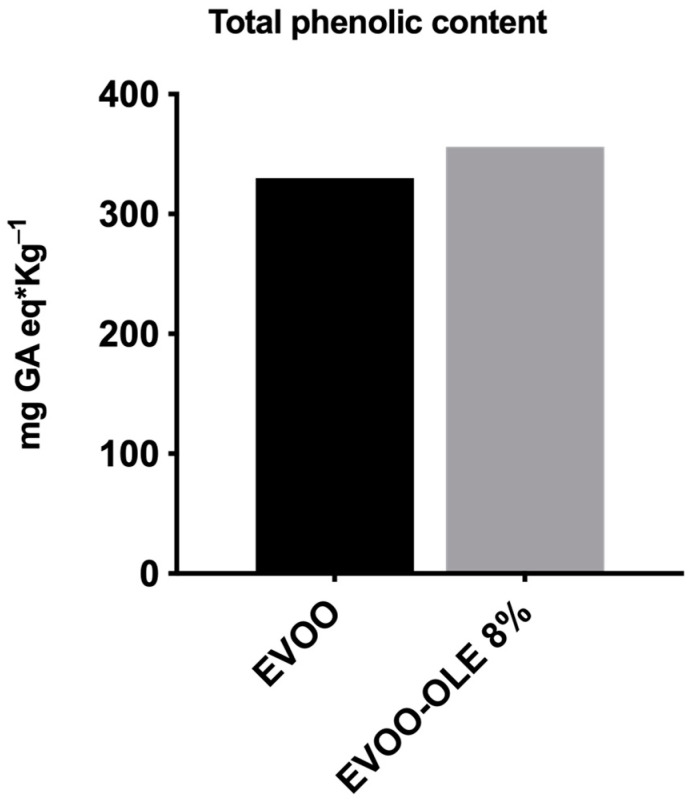
Total phenolic content (TPC) of EVOO and EVOO/OLE 8% extracts.

As reported in Figure 2, TPC concentration of EVOO is 330 mg GA eq/Kg oil. The addition of 8% OLE to the EVOO extract provided an increase in TPC, presenting a new value of 360 mg GA eq/kg oil. Notably, the total phenolic content of OLE is 58.47 mg GA equivalents/g of OLE. It is worth noting that the addition of OLE caused a 10% increase in TPC, confirmed by the HPLC phenolic characterization.

Considering the role of these polyphenols in the influence of antioxidant and anti-inflammatory properties, EVOO/OLE 8% have been further investigated.

### 3.2. Antioxidant Activity

Several assays have recently been developed to evaluate the antioxidant capacity of foods. These methods differ both on the basis of antioxidant measurements, which include the formation of different radicals and/or the detection of reduced metal cations, and on how end points are measured. Moreover, considering that the antioxidant compounds may act in vivo through different mechanisms and that quite often the antioxidant effect is due to a combination of the actions of diverse antioxidant polyphenols, a single method can be inadequate to completely evaluate the antioxidant capacity of food [45]. Three in vitro established antioxidant systems (DPPH, ABTS, and FRAP assays) were used to evaluate the antioxidant capacities of OLE, EVOO, and the enriched EVOO/OLE 8% extracts.

The DPPH is normally considered a radical scavenging assay and is one of the most frequently employed single electron transfer-based antioxidant procedures because of its ease of performance, rapidness, automation potential, reproducibility, and usability at ambient temperature [46]. EVOO, OLE, and EVOO/OLE 8% samples were tested in the proper range of concentration, reporting a dose response relationship. The EC_50_ values for DPPH scavenging of all three extracts are reported in Table 2. As expected, OLE showed a good ability to reduce DPPH radical scavenging with an EC_50_ of 0.147 mg/mL (Table 2). The EVOO/OLE 8% extract demonstrated an improvement in radical scavenging activity compared to the EVOO extract. In fact, the EC_50_ of EVOO/OLE 8% is 11 times greater than that of single EVOO. Following the addition of only 8% of OLE, the antioxidant capacity was significantly increased.

The ABTS assay measures the efficiency in scavenging the radical cation ABTS•^+^, which was reduced to ABTS, and the data are reported as the efficient concentration to decrease the initial ABTS concentration by 50% (EC_50_). Similar to DPPH results, OLE reported the strongest antioxidant effects (EC_50_ = 0.055 mg/mL), while the EC_50_ of EVOO/OLE 8% showed an increase of 2-fold with respect to EVOO extract.

The FRAP assay shows a different mechanism of action since it does not implicate a reaction regarding free radicals but rather evaluates the ability to reduce ferric ions (Fe^3+^) to ferrous ions (Fe^2+^) [47]. The FRAP value of samples ranged from 1.90 to 2.90 mmol Trolox/kg using this assay. Even in this assay, the addition of OLE conferred improved antioxidant activity to EVOO extract, reporting an enhancement of ferric reducing ability compared to the antioxidant activity of EVOO (Table 2). This improvement is quite interesting because the redox ability of OLE (1.90 mmol Trolox/kg) is similar to that of EVOO (2.55 mmol Trolox/kg).

These results highlight the importance of using different methods to assess the antioxidant activity of an extract. In fact, as already discussed [48], the evaluation of different processes such as radical scavenging activity (DPPH and ABTS) and redox reducing ability (FRAP) offers a complete panel of fundamental antioxidant processes.

### 3.3. Anti-Inflammatory Profile

In the present study, OLE, EVOO, and EVOO/OLE 8% extracts have been evaluated for their ability to inhibit the catalytic activities of COX-1 and COX-2. The assay allowed the measurement of the enzymatic activity of the peroxidase component of COXs, through the use of a commercial colorimetric kit (Cayman Chemicals). The method was based on the detection of the oxidation of the chromogenic substrate N,N,N′,N′-tetramethyl-*p*-phenylenediamine (TMPD) at λ = 590 nm.. The catalytic activities of the two enzymes were quantified by measuring TMPD formation in the presence of various concentrations of the test compounds, revealing a dose-response inhibition.

**Figure 3:**
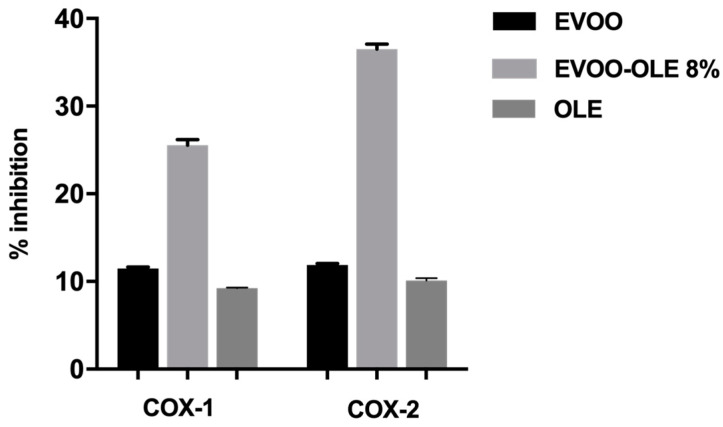
Anti-inflammatory effect at 225 µg/mL of EVO and EVOO/OLE 8% extracts.

OLE, EVOO, and EVOO/OLE 8% extracts were initially tested at 225 µg/mL, evaluating an improved COX-1 and COX-2 anti-inflammatory effect for the enriched EVOO/OLE 8% compared to the EVOO extract and OLE, as reported in Figure 3.

The percentage of COX-1 inhibitory activity at 225 µg/mL resulted of 11.5% for EVOO extract, 9.25% for OLE, and 25.5% for EVOO/OLE 8% (Figure 3). Likewise, the percentage of COX-2 inhibitory activity at 225 µg/mL was 11.9% for EVOO extract, 10.1% for OLE, and 36.5% for EVOO/OLE 8%.

For both enzymes, although OLE and EVOO extract demonstrated about 10% inhibition, the EVOO/OLE 8% extract reported more than 25% activity with an enhancement of about 3-fold.

Regarding IC_50_ data, the EVOO/OLE 8% IC_50_ value (IC_50_ COX-1 = 0.475 mg/mL; IC_50_ COX-2 = 0.383 mg/mL, Table 3) is improved by 4-fold in COX-1 inhibition and 2-fold in COX-2 inhibition with respect to the IC_50_ of the EVOO extract (IC_50_ COX-1 = 1.90 mg/mL; IC_50_ COX-2 = 0.90 mg/mL, Table 3) as reported in Table 3.

## 4. Discussion

EVOO represents a functional food with health properties mainly attributed to the presence of phenolic compounds. The most abundant phenolic compounds in EVOO, endowed with nutraceutical properties, are the secoiridoids: oleocanthal and oleacein, and the simple alcohols: tyrosol and hydroxytyrosol. Oleuropein is a secoiridoid with several beneficial health properties and is the most representative polyphenol in olive leaves, a waste by-product derived from the harvesting of olive trees, but unfortunately it is poorly present in EVOO [23]. Therefore, the addition of oleuropein-rich OLE to EVOO extract could represent a high value for EVOO, using oleuropein enrichment in order to improve its nutraceutical properties. The advantage in terms of nutraceutical properties for the EVOO/OLE extract thus obtained could be further enhanced due to beneficial synergistic interactions between the different polyphenols [32]. In this work, we evaluated some extracts obtained by adding different percentages of oleuropein-rich OLE to an EVOO extract. From the qualitative and quantitative study of these extracts, performed by HPLC analysis, we were able to select the EVOO/OLE 8% extract, which has a balanced quantity of the most representative polyphenols (oleocanthal, oleacein, and oleuropein) of almost 10 mg/g of extract, as a starting point to investigate a hypothetical synergistic effect as already reported in other studies [49,50]. The high content of oleacein, oleocanthal, and oleuropein in the 8% extract of EVOO/OLE 8% allowed us to evaluate how the biological activity might be influenced by the interaction of nutraceutical polyphenols.

The EVOO/OLE 8% extract was subjected to further investigations to evaluate its antioxidant and anti-inflammatory properties.

The antioxidant effect is fundamental as a nutraceutical property of a food extract, as it delays oxidative processes and can contribute to the prevention of numerous chronic diseases [51]. EVOO has antioxidant properties that are linked to polyphenols like oleacein and oleocanthal [52]. However, oleuropein, contained in olive leaves, is characterized by strong antioxidant activity, particularly as a free radical scavenger [53]. In this study, we investigated the antioxidant activity in terms of the evaluation of the radical scavenging effect on the DPPH• and ABTS•^+^ radicals of OLE, EVOO, and EVO/OLE 8% extracts. In both in vitro assays (DPPH and ABTS) used to evaluate the antioxidant capacity, the EVOO/OLE 8% extract displayed an increased antioxidant effect compared to EVOO extract, demonstrating the additional antioxidant action of oleuropein. As expected, OLE reported a high EC_50_ value, as demonstrated by the literature, but it should be noted that with only the 8% of OLE addition, the antioxidant effect of EVOO/OLE 8% is significantly improved over EVOO extract.

It is well known that the antioxidant activity could be due to a combination of different mechanisms; for this reason, we tested OLE, EVOO, and EVOO/OLE 8% extracts also for their redox ability to reduce ferric ions by the FRAP assay. In this assay, all the extracts demonstrated a similar activity, but the improvement of the EVOO/OLE 8% antioxidant effect compared to EVOO extract and OLE was confirmed.

In addition to oxidative stress, several studies assert the role of inflammation in the onset of a wide variety of age-related disorders such as diabetes, cardiovascular disease, cancer, central nervous system-related and autoimmune diseases [54,55]. As previously mentioned, oleocanthal possesses anti-inflammatory properties as it is able to inhibit inflammatory mediators COX-1 and COX-2 similarly to ibuprofen, a known anti-inflammatory drug [8]. Moreover, recently, the anti-inflammatory activity of oleacein in terms of COX inhibition was reported [16]. Consequently, in this study, we investigated the anti-inflammatory properties of oleocanthal-rich EVOO, OLE, and EVOO/OLE 8% extracts, evaluating their capability to inhibit COX-1 and COX-2 enzymes. Preliminarily, all the extracts have been evaluated at 225 μg/mL. The percentage of inhibition reported by OLE and EVOO for both enzymes was quite similar, close to 10% of inhibition (Figure 3). Surprisingly, the EVOO/OLE 8% extract reported a 25.5% COX-1 inhibition and a 36.5% COX-2 inhibition (Figure 3). The interaction among the polyphenols presented in EVOO extract and OLE significantly affects the inhibition of these proinflammatory enzymes.

The high presence of the three most important polyphenols (oleocanthal, oleacein, and oleuropein) in EVOO/OLE 8% extract gave a positive contribution to the antioxidant and anti-inflammatory properties compared to EVOO extract.

It is very important to underline that a small addition of OLE to EVOO extract, which does not much affect the total amount of polyphenols, significantly ameliorates the nutraceutical properties of EVOO.

## 5. Conclusions

In this study, we investigated EVOO/OLE extracts obtained by adding different percentages of oleuropein-rich OLE to the EVOO extract. The extracts thus obtained are enriched with oleuropein, maintaining a high level of oleocanthal and oleacein typical of EVOO. This addition was found to be significant in terms of nutraceutical properties, as the newly selected EVOO/OLE 8% extract demonstrated improved antioxidant and anti-inflammatory properties compared to the singular EVOO extract.

This new approach represents a strategy for the further enhancement of olive leaves as by-products of great economic and environmental value.

## Figures and Tables

**Table 1:** Phenolic compound contents (mg/g of extract) of EVOO/OLE, EVOO and OLE.

Phenolic Compounds	EVOO/OLE				EVOO	OLE
	EVOO/OLE 8%	EVOO/OLE 4%	EVOO/OLE 2%	EVOO/OLE 1%		
tyrosol	0.99 ± 0.058	0.89 ± 0.097	0.92 ± 0.016	0.95 ± 0.006	1.02 ± 0.04	
hydroxytyrosol	0.55 ± 0.029	0.51 ± 0.015	0.49 ± 0.01	0.56± 0.033	0.72 ± 0.025	
oleacein	10.84 ± 0.72	10.77 ± 0.097	10.02 ± 0.46	10.45 ± 0.097	11.05 ± 0.04	
oleocanthal	19.03 ± 0.42	19.98 ± 1.16	20.29 ± 0.16	19.65 ± 0.66	20.97 ± 0.89	
oleuropein	8.46 ± 0.16	2.75 ± 0.003	1.55 ± 0.064	0.80 ± 0.026		35.58 ± 2.21
luteolin-7-*O*- glucoside	1.80 ± 0.15	0.48 ± 0.028	0.23 ± 0.016	0.13 ± 0.011		6.70 ± 0.61
apigenin 7-*O*- glucoside	0.65 ± 0.019	0.25 ± 0.021	0.20 ± 0.001	0.15 ± 0.007		1.80 ± 0.035

**Table 2:** Antioxidant capacity of EVOO and EVOO/OLE 8% measured by DPPH, ABTS, and FRAP assays.

Sample	DPPH ^a^ EC_50_ mg/mL	ABTS ^b^ EC_50_ mg/mL	FRAP ^c^ mmol Trolox/kg
EVOO	5.55 ± 0.29	1.21 ± 0.13	2.55 ± 0.006
EVOO/OLE 8%	0.50 ± 0.001	0.419 ± 0.04	2.90 ± 0.08
OLE	0.147 ± 0.001	0.055 ± 0.003	1.90 ± 0.04

^a^ DPPH: radical scavenging activity assay; ^b^ ABTS: radical scavenging activity assay. ^c^ FRAP: ferric reducing ability power assay. ^b^ Values are the average of three determinations ± standard deviation. Different letters in the same column indicate a significant difference (Turkey test *p* > 0.05).

**Table 3:** Inhibitory effects of EVOO and EVOO/OLE 8% on COX-1 and COX-2.

Sample	COX-1 IC_50_ mg/mL	COX-2 IC_50_ mg/mL
EVOO	1.90 ± 0.17	0.9 ± 0.01
EVOO/OLE 8%	0.475 ± 0.06	0.383 ± 0.009

Values are the average of three determinations ± standard deviation.

## Data Availability

Not applicable.
